# Iron ochre – a pre-catalyst for the cracking of methane

**DOI:** 10.1002/jctb.4434

**Published:** 2014-06-26

**Authors:** Abdulrahman Alharthi, Ross A Blackley, T Hugh Flowers, Justin S J Hargreaves, Ian D Pulford, James Wigzell, Wuzong Zhou

**Affiliations:** a School of Chemistry, Joseph Black Building, University of GlasgowGlasgow, G12 8QQ, UK; b School of Chemistry, Purdie Building, University of St AndrewsSt Andrews, KY16 9ST, UK

**Keywords:** hydrogen, iron ochre, biogenic iron oxide, carbon, *leptothrix*

## Abstract

**BACKGROUND:**

Iron ochres are gelatinous sludges that can cause problems in terms of water management. In this work, the application of iron ochre obtained from a river has been applied to catalytically crack methane – another potential waste product – into two useful products, hydrogen and a magnetic carbon-containing composite.

**RESULTS:**

The powder X-ray diffraction (XRD) pattern of the iron ochre was found to be consistent with the expected 2-line ferrihydrite, and energy dispersive X-ray (EDX) analysis showed Fe to be a major component although some Si and Ca were present. The sample was observed to contain a fraction with a tubular morphology consistent with the presence of extra-cellular biogenic iron oxide formed by *leptothrix*. Upon exposure to methane at elevated temperatures, the material was found to transform into an active catalyst for hydrogen production yielding a magnetic carbon-containing composite material comprising filamentous carbon and encapsulating graphite.

**CONCLUSION:**

The application of two waste products – iron ochre and methane – to generate two useful products – hydrogen and a magnetic carbon-containing composite – has been demonstrated. Furthermore, the ochre has been shown to comprise tubular morphology extra-cellular biogenic iron oxide which may be of interest in terms of other applications. © 2014 The Authors. *Journal of Chemical Technology & Biotechnology* published by John Wiley & Sons Ltd on behalf of Society of Chemical Industry.

## INTRODUCTION

The catalytic cracking of methane to yield hydrogen and carbon is a reaction of current interest presenting a possible route to CO_x_-free H_2_ which is, for example, desirable for application in PEM fuel cells where there is a high susceptibility to poisoning by trace levels of CO. The reaction also presents a route towards carbon nanostructures, such as carbon nanotubes. Within the literature, a number of catalysts have been identified as being effective, including those containing Ni, Co, Pd and Fe.^1^–^3^ The carbon-containing composite products may find further application, for example Fe–C-containing composites have been investigated for water treatment (where advantage may be taken of their magnetic nature, leading to improved ease of separation)^4^ and use in demulsification of oil/water mixtures.^5^

The focus of this study is the investigation of iron ochre as a potential active material for methane cracking. Iron ochres are commonly occurring gelatinous sludges which are formed when ferrous ions within bodies of water become oxidized. They occur in many aqueous environments, including rivers, waste waters from mining operations, in domestic water systems and in field drainage. In some instances they prove problematic, sometimes blocking drainage systems completely.^6^ Ochres therefore represent a prevalent waste resource and they have been studied for a number of applications including their use as pigments and as materials for water treatment. However, it seems that they have seldom, if ever, been studied for catalytic applications. This is perhaps somewhat surprising in view of the current increasing interest in the application of waste materials for catalytic purposes.^7^

In this study, the application of iron ochre occurring in a river to the cracking of methane to generate hydrogen and a magnetic carbon-containing composite is detailed. Methane, a potent greenhouse gas, can also be considered a waste from refinery operations, where it is flared, and from landfill dumping. This study therefore represents the valorisation of two large-scale wastes – iron ochre and methane – to produce two desirable products, H_2_ and a carbon-containing magnetic composite. A similar approach has previously been applied with red mud, another iron-containing large-scale waste.^8^,^9^

## EXPERIMENTAL

The reactor and procedures employed for catalytic activity determination were as reported previously.^8^,^9^ In summary, materials were used in the form of powders held centrally within the heated zone of a quartz microreactor between quartz wool plugs. The feed gas composition applied was a mixture of 75% methane and 25% nitrogen (BOC, 99.98%) flowing at a total rate of 12 mL min^−1^ over *ca*. 0.4 g material charge. Product analysis was performed by on-line gas chromatography (GC) for H_2_ quantification (Hewlett Packard 5890A) using a TCD for detection and a 12′ long and 1/8″ O.D. Molecular Sieve 13X packed column for product separation. Calibration was undertaken using a series of H_2_-containing mixtures of known composition. In addition, CO*_x_* quantification was undertaken by Fourier transform infrared (FTIR) analysis of aliquots of the effluent reactor stream which were sampled by passage through a gas-phase FTIR cell. FTIR analysis was undertaken using a Jasco 4100 FTIR spectrometer operating in the 400–4000 cm^−1^ spectral range acquiring 64 scans for each spectrum at a resolution of 4 cm^−1^ following background subtraction. Calibration was performed using a multicomponent mixture of known CO and CO_2_ concentration. A limitation of this method is that it becomes insensitive to CO_2_ levels below those used for the background subtraction. Data were calculated as mass normalized rates corresponding to the mass of iron ochre charged to the reactor and the effects of molar expansion upon reaction were normalized with reference to the nitrogen internal standard.

The samples were characterized by powder X-ray diffraction (XRD), scanning electron microscopy (SEM), high resolution transmission electron microscopy (HRTEM), CHN analysis, thermogravimetric analysis (TGA) in air and BET surface area analysis. XRD measurements were performed using a Siemens D5000 diffractometer with Cu Kα radiation. A 2θ range between 5 and 85° was scanned using a counting rate of 1 s per step with a step size of 0.02° and samples were prepared by compaction into a sample holder. SEM was undertaken in an XL30 ESEM Phillips microscope operating at 25 kV. Samples were dispersed on a carbon stub and were coated using a Polaron SC7640 Auto high resolution sputter coater with a gold/palladium target. HRTEM was performed on a JEM-2011 electron microscope fitted with a LaB_6_ filament, with a point resolution of 1.8 Å. Samples were dispersed in acetone and dropped onto holey carbon grids. CHN was determined by combustion using an Exeter Analytical CE-440 elemental analyzer. TGA was performed using a TA Instruments QA instrument with measurements being undertaken in the temperature range from ambient to 1000 °C at a heating rate of 10 °C min^−1^ employing 8 mg of sample and a flow rate of air of 50 mL min^−1^. BET surface areas were measured with a Miromeritics Gemini instrument using N_2_ physisorption at liquid nitrogen temperature (−196 °C) following appropriate degassing of samples.

## RESULTS AND DISCUSSION

**Figure 1:**
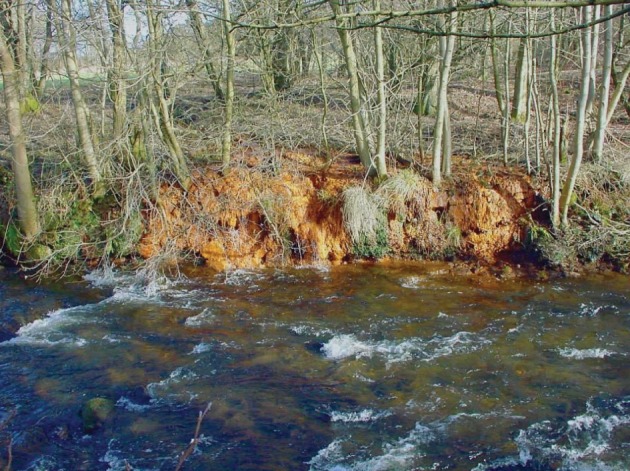
The site in the River Allander from where the ochre was obtained.

**Figure 2:**
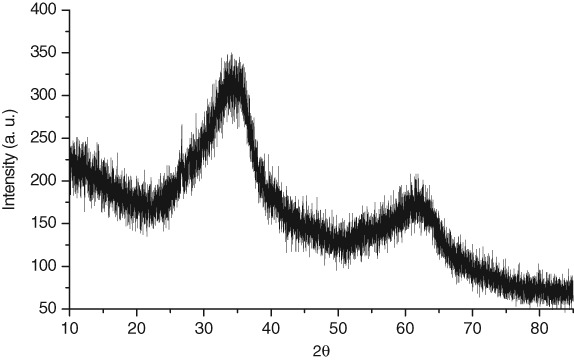
Powder X-ray diffraction pattern of the pre-reaction iron ochre.

**Figure 3:**
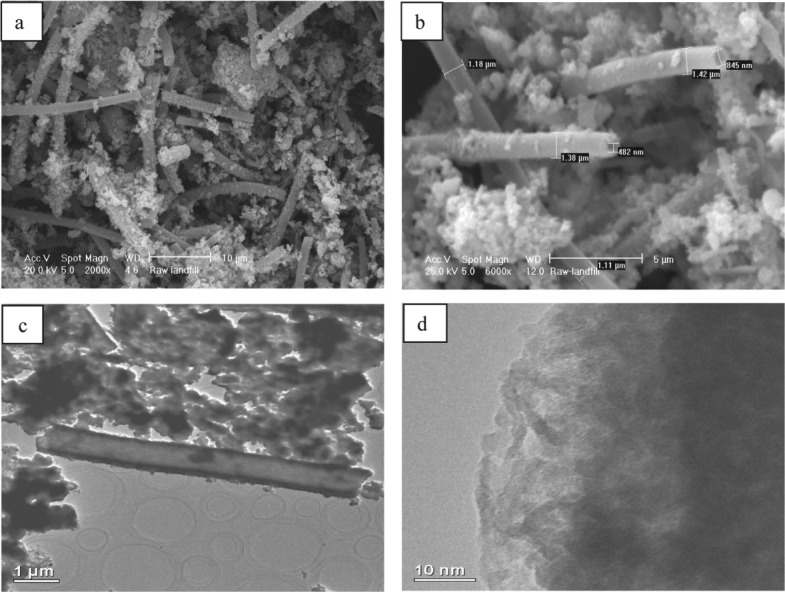
(a) and (b) SEM micrographs of the pre-reaction iron ochre (c) TEM demonstrating the tubular morphology and (d) TEM micrograph of a tube wall.

**Figure 4:**
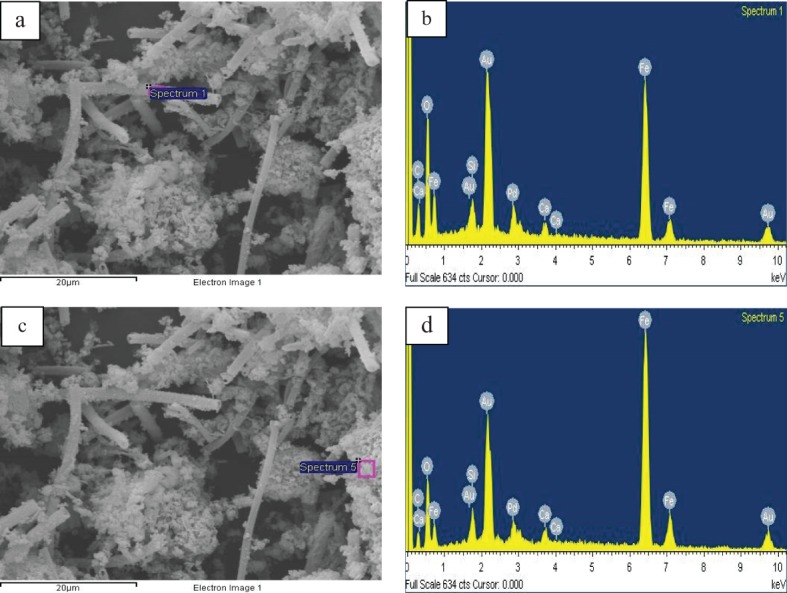
SEM and EDX spectra of the iron ochre sample: (a) position of EDX analysis (b) for a microtube; (c) position of EDX analysis (d) of the irregular morphology material. The Au and Pd spectral features relate to the sputter coating of the samples.

The iron ochre investigated in this study was harvested from the River Allander in Milngavie, a town close to Glasgow in the United Kingdom. The Ordnance Survey Grid Reference for the point of sampling is NS 54711 75743. Figure 1 is a photograph of the site in which the characteristic orange/red colouration of the ochre associated with the river bank can be seen. A sample of the ochre was taken from the river and, following removal of debris such as twigs, was thoroughly washed and dried in a laboratory oven at 110 °C. As shown in Fig. 2, powder X-ray diffraction undertaken on the sample shows it to be generally X-ray amorphous with two broad reflections centred at *c*. 35 and 60° (2θ) being apparent, corresponding to 2-line ferrihydrite, an amorphous iron oxyhydroxide.^10^ The material also possesses a large BET surface area (254 m^2^ g^−1^) suggesting it to be highly dispersed and/or porous. Accordingly, SEM investigation was undertaken and representative micrographs are shown in Fig. 3(a) and (b). The results are striking. Two distinct morphological components are present, an irregular fraction and a fraction comprising apparently hollow tubes of various lengths and diameters in the micrometre size range. This tubular component is characteristic of extracellular biogenic iron oxide coating the bacterium *leptothrix*.^11^–^13^ These tubes may contain a dead bacterial cell. Energy dispersive X-ray (EDX) spectra were taken of the various regions of the ochre sample and representative results are shown in Fig. 4. The elemental compositions of the irregular and tubular regions of the sample were found to be broadly similar with an atomic ratio of *ca*. 93:4:3 for Fe:Si:Ca. CHN analysis showed the sample to contain 4.52 +/− 0.02 wt% carbon which, at least in part, could relate to the bacterial content of the sample. HRTEM investigation of the biogenic microtubes was undertaken, as shown in Fig. 3(c) and (d) and, consistent with the literature, they were shown to be comprised of irregular morphology material.

**Figure 5:**
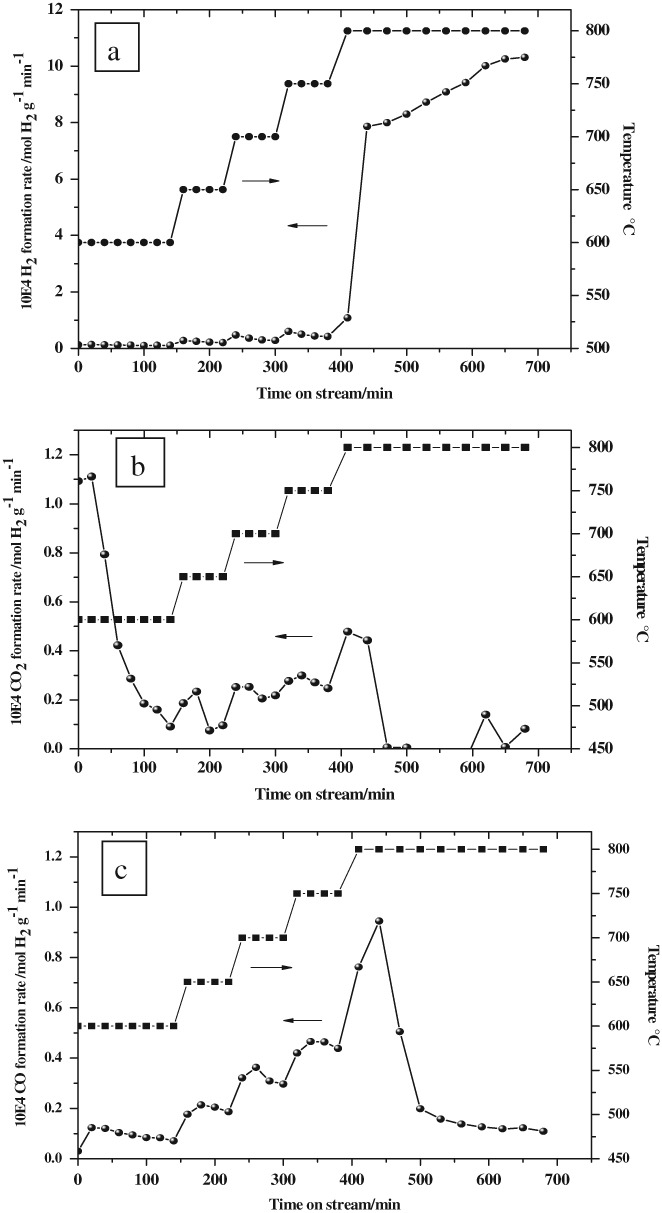
Temperature programmed reaction results for the iron ochre (a) mass normalized H_2_ formation rate, (b) mass normalized CO_2_ formation rate and (c) mass normalized CO formation rate.

The material was tested to determine its methane cracking activity. The hydrogen formation rate in a temperature programmed experiment between 600 °C and 800 °C is shown in Fig. 5(a). Corresponding CO_x_ data are shown in Fig. 5(b) and (c). It can be seen that hydrogen production is enhanced with each increment in temperature and at 800 °C there is a delayed stepped increase in formation rate. Pressure drop effects (due to carbon deposition) occurred at prolonged times on stream beyond the step in activity. It is also apparent that below 800 °C, temperature increments are associated with initial maxima in H_2_ formation rate, followed by gradual decline, possibly corresponding to stepwise transformation processes. CO_2_ formation is maximal at the lowest temperature in the temperature programme (600 °C) and then it decays with time on stream, although temperature programming led to maxima which reduced in intensity as the temperature was increased. At extended times on stream at 800 °C, CO_2_ declines (although the limitations of the FTIR technique in relation to the background CO_2_ levels should be kept in mind meaning that the measurement is not reliable for quantification of low levels of CO_2_). The CO profile is somewhat different from its CO_2_ counterpart in that there is an evident increase as a function of temperature. At 800 °C, a peak formation rate is observed which decays just before the peak in the H_2_ formation rate. It should be noted that H_2_ free from the presence of CO is not formed in this experiment. This could be a consequence of incomplete reduction. In many studies in the literature, pre-reduction of materials applied to methane cracking is undertaken with little consideration being given to hydrogen balances (i.e. the quantity of hydrogen applied for reduction versus that recovered from cracking.) However, in terms of the combination of two waste products (iron ochre and methane), the omission of a pre-reduction step is more appropriate. The general form of the profile is reproducible, but since ferrihydrite is a metastable phase, changes can occur as a function of storage time, which can influence the times on stream necessary to attain maximum hydrogen formation rate.

**Figure 6:**
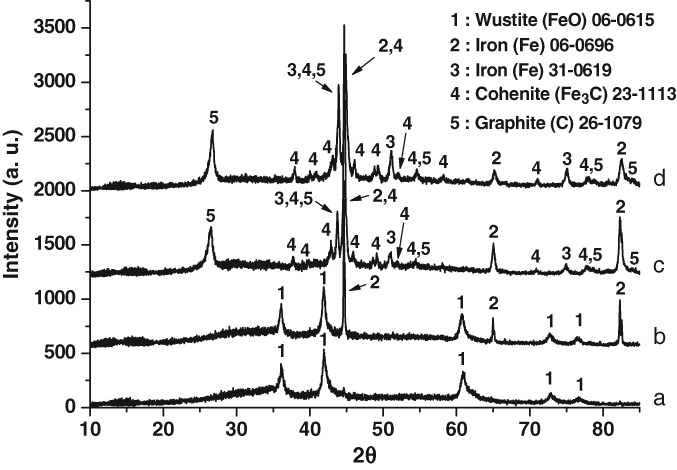
Powder X-ray diffraction patterns of the iron ochre following reaction with methane for (a) 600 °C (15 h on stream), (b) 700 °C (15 h on stream), (c) 750 °C (12 h on stream) and (d) 800 °C (11.5 h on stream)

The form of the reaction profiles presented are generally consistent with other studies in which the reaction of methane with non-reduced iron oxide-hydroxide phases has been investigated.^14^ The processes prior to the main burst of H_2_ at 800 °C correspond to stepwise reduction processes which produce H_2_, CO and CO_2_ as reaction products in pathways such as:

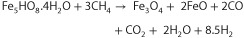


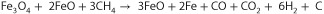


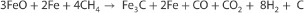


with the oxidic iron phases functioning as pre-catalysts to catalytically active Fe and Fe_3_C. The equations shown above are not intended to be exact, but are rather illustrative of potential stepwise reduction processes to generate the catalytically active phases. Fe_5_HO_8_.4H_2_O represents the formula for ferrihydrite.^10^ Support for the sequential reduction of ferrihydrite through intermediate oxidic phases is provided by phase analysis of XRD patterns of ochre samples reacted with methane at different temperatures as shown in Fig. 6.

**Figure 7:**
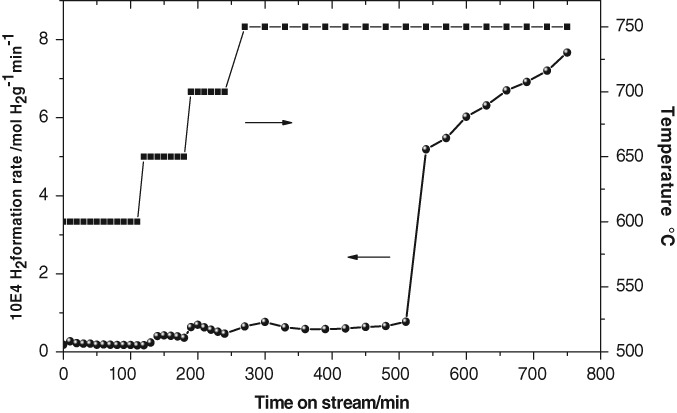
H_2_ formation rate profile for the temperature programmed reaction of iron ochre with methane up to 750 °C.

At a critical point of reduction, Fe and/or Fe_3_C is formed which then catalytically cracks methane generally resulting in deactivation fairly rapidly due to carbon deposition. At lower temperatures, this reduction process occurs at longer times on stream, as demonstrated by the H_2_ formation profile in Fig. 7 where a significant delay in onset is apparent at the lower maximum reaction temperature of 750 °C.

**Figure 8:**
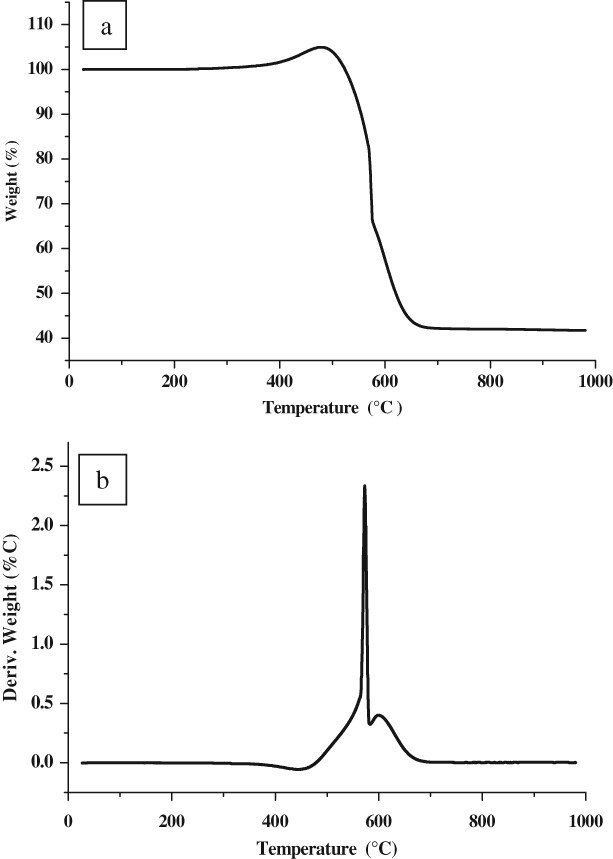
(a) Temperature programmed oxidation TGA profile under air for post-reaction iron ochre, and (b) corresponding first derivative.

**Figure 9:**
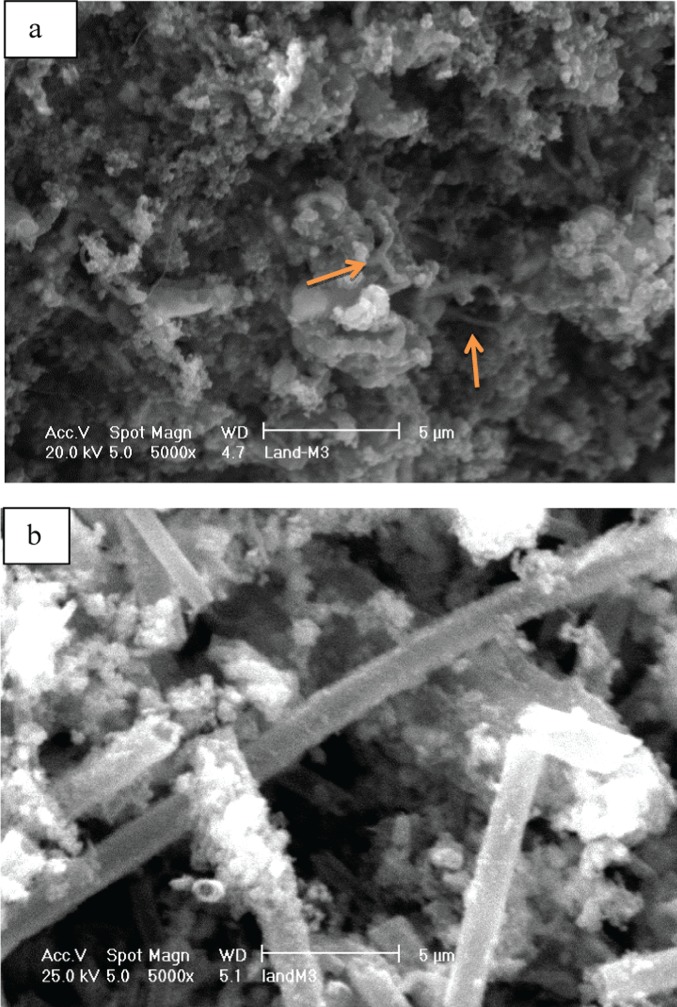
SEM micrographs of the post-reaction iron ochre sample: (a) SEM micrograph shows the formation of carbon nanofibres, indicated by arrows; (b) SEM micrograph shows the remnants of the tubular morphology in the sample.

**Figure 10:**
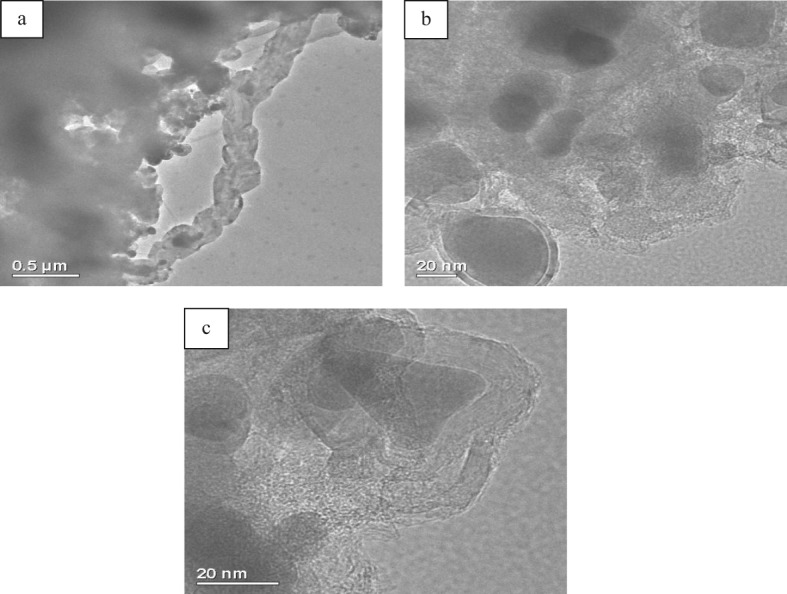
TEM micrographs of the post-reaction iron ochre sample: (a) TEM micrograph shows the formation of chain-like carbon nanofibres with iron related phases at the centre; (b) and (c) TEM micrographs showing the formation of graphite enclosed Fe and/or Fe_3_C.

Post-reaction XRD analysis was undertaken on the sample subjected to the temperature programmed reaction shown in Fig. 6. The reflections evident can be matched to Fe, Fe_3_C and graphite. A simple test using a bar magnet demonstrated the material to be magnetic. Post-reaction CHN analysis demonstrated this sample to contain 64.91 +/− 0.90 wt% C and there was a drastic reduction in surface area to 41 m^2^ g^−1^. Temperature programmed oxidation of the sample under air was performed and the TGA profile is presented in Fig. 8(a). Before the major mass loss, corresponding to oxidation of the post-reaction carbon, the sample gained mass as a result of the oxidation of reduced iron phases as has been observed in related studies.^8^,^9^ The inflection in the mass loss profile is reproducible and in order to see it more clearly, the derivative mass loss profile is shown in Fig. 8(b). This profile is indicative of the possible presence of two carbon-containing species possessing maxima in their rate of oxidation at *ca*. 580 and 600 °C, the former showing an apparently narrower distribution of reactivity. In order to investigate these species in more detail, the sample was investigated by SEM and the results are presented in Fig. 9. Overall, a major change in morphology has occurred although some filamentous carbon is present as indicated in the micrographs in Fig. 9(a). Some remnant of the original tubular morphology was observed in the post-reaction sample, Fig. 9(b), although it should be emphasized that this was a very minor component. TEM observations also evidence filamentous carbon (Fig.10(a)) and graphite enclosed Fe and/or Fe_3_C as shown in Fig.10(b) and (c). Such features are common for carbons formed over iron-containing materials under comparable reaction conditions.^15^ It is tempting to ascribe the two TGA mass loss features to these two morphologies. The potential catalytic influence of the iron phases in oxidizing the carbon should be kept in mind, along with the apparently broad distribution of species oxidized around the maximum at 600 °C suggesting a wide range of reactivity.

Compared with a red mud sample investigated previously^8^,^9^ and run under comparable reaction conditions, the iron ochre sample showed an earlier on-set of activity with a higher maximum formation rate. It is believed that this corresponds to the higher iron content in the ochre along with the lower concentration of sodium, which is an acknowledged poison for methane cracking.^14^

A potential concept arising from this study which has previously been proposed for iron- containing catalysts,^16^ is the use of iron ochre to facilitate the transportation of methane over long distances. Methane is often found in remote locations where its transportation costs prohibit its further application. However, it can be decomposed over iron ochre, to yield a valuable hydrogen-containing product gas, generated from two wastes, and a carbonaceous deposit which could be gasified to yield valuable syn gas in another location following transportation.^16^

Another interesting aspect of this study is the potential route to micronscale tubes of Fe and/or Fe_3_C, which could be interesting materials in their own right. Ongoing studies are currently being directed towards this prospect; both employing more reactive reductants to lower the temperature of transformation and/or dopants more effective at activation of reductants which can provide loci for spillover of activated reductant species.

## CONCLUSION

This study has demonstrated the production of two useful products, hydrogen and a magnetic carbon-containing composite, from two wastes – iron ochre comprising biogenic extracellular 2-line ferrihydrite, and methane. However, under the conditions studied, the H_2_ produced contains CO. The carbon produced is in the form of filaments and graphite encapsulating reduced iron species and it may have downstream application in enhancing separation and/or water pre-treatment. This represents one potential application for iron ochres which in some circumstances impede water flow necessitating their removal and disposal.
